# Attachment Representations and Early Interactions in Drug Addicted Mothers: A Case Study of Four Women with Distinct Adult Attachment Interview Classifications

**DOI:** 10.3389/fpsyg.2016.00346

**Published:** 2016-03-16

**Authors:** Alessio Porreca, Francesca De Palo, Alessandra Simonelli, Nicoletta Capra

**Affiliations:** ^1^Department of Developmental and Social Psychology, University of PaduaPadua, Italy; ^2^Therapeutic Community “Casa Aurora” – Comunità di Venezia s.c.s.Venice, Italy

**Keywords:** parenting, attachment representations, mother-child interactions, drug addiction, child development

## Abstract

Drug addiction is considered a major risk factor that can influence maternal functioning at multiple levels, leading to less optimal parental qualities and less positive interactive exchanges in mother-child dyads. Moreover, drug abusers often report negative or traumatic attachment representations regarding their own childhood. These representations might affect, to some extent, later relational and developmental outcomes of their children. This study explored whether the development of dyadic interactions in addicted women differed based on attachment status. The longitudinal ongoing of mother-child emotional exchanges was assessed among four mothers with four different attachment statuses (F-autonomous, E-preoccupied, Ds-dismissing, and U-unresolved/with losses). Attachment representations were assessed using the Adult Attachment Interview (George et al., 1985), while mother-child interactions were evaluated longitudinally during videotaped play sessions, through the Emotional Availability Scales (Biringen, 2008). As expected, the dyad with the autonomous mother showed better interactive functioning during play despite the condition of drug-abuse; the mother proved to be more affectively positive, sensitive, and responsive, while her baby showed a better organization of affects and behaviors. On the other side, insecure mothers seemed to experience more difficulties when interacting with their children showing inconsistency in the ability to perceive and respond to their babies' signals. Finally, children of insecure mothers showed less clear affects and signals. While differences between secure and insecure dyads appeared clear, differences between insecure patterns where less linear, suggesting a possible mediating role played by other factors. Clinical implications and suggestions for future research are discussed.

## Introduction

### The complex system of parenting

Parenting could be defined as a complex function, determined by multiple aspects, which has to do with the ability to take care of someone else, to provide them nurturance and to interpret correctly their needs and accept their subjectivity under different conditions (Belsky, 1984; Fava Vizziello, 2003; Simonelli, 2009, 2014). This ability is influenced both by past experiences (through mental representations and expectations built during childhood) and by actual experiences with the baby (lived in everyday interactions).

From an attachment perspective, parenting could be conceptualized as an organized behavioral system, goal corrected and maintained through internal working models (Bowlby, 1988; Solomon and George, 1996). Part of these processing systems are based on the individuals' evaluation of their childhood experiences which are thought to be organized in “states of mind” relatively stable with respect to attachment (Main et al., 1985). These representations regulate affects in a predictable manner and determine the adults' sensitivity and responsiveness to infant signals, shaping the quality of adult-child attachment (Kaplan, 1984; Main et al., 1985; Bretherton et al., 1990; Solomon et al., 1995; Van Ijzendoorn, 1995; George and Solomon, 1996). It has been shown that sensitive parents are more likely to have children with secure attachments while non-sensitive or non-responsive parents are more likely to have children insecurely attached to them (Ainsworth et al., 1978; Smith and Pederson, 1988; Isabella and Belsky, 1991; Isabella, 1993; Van Ijzendoorn et al., 1995).

The link between adult attachment representations, parental sensitivity and attachment security resulted moderate anyway, suggesting a “gap” in the intergenerational transmission of attachment and the consequent need to take into account other relational features in order to better understand parental functioning and the nature of early adult-child relationships (Van Ijzendoorn, 1995; van Ijzendoorn and Bakermans-Kranenburg, 1999; Biringen et al., 2014; Porreca et al., 2015).

As an expansion upon the original conceptualization of the parent-child attachment relationship, emotional availability appears a particularly useful concept in this sense (Biringen and Robinson, 1991; Biringen et al., 1994; Saunders et al., 2015); in fact, it recalls in part sensitivity as referred to by attachment theorists but at the same time adopts a wider perspective, emphasizing the “emotional features” of adult child-interactions, intended both as the ability of the parent to adequately signal and to correctly perceive infant emotional bids (Biringen et al., 2000, 2014; Biringen and Easterbrooks, 2012). Beyond sensitivity, emotional availability focuses on other aspects of adult behaviors (structuring, non-intrusiveness and non-hostility) and gives equal importance to the child's contribution, considering his/her ability to respond to the parent and to appropriately involve him/her during interactions (Biringen and Easterbrooks, 2012). Each of these aspects is read in a dyadic way, keeping in mind contextual aspects.

### Drug addiction and parenting

Drug addiction and substance abuse constitute severe risk factors for maternal functioning and for the child's psychological and physical health (Klee et al., 2002; Barnard and McKeganey, 2004; Berlin et al., 2008). The prolonged use of substances during pregnancy and the often associated avoid of the required medical screenings increase the risk for prematurity and reduced growth measures at delivery (Di Cagno et al., 1985; Zuckerman et al., 1989; Zuckerman and Bresnahan, 1991; Mayes et al., 1992; Malagoli Togliatti and Mazzoni, 1993; Hunter and Powis, 1996; Fava Vizziello et al., 1997; Bona and Zaffaroni, 2003).

Once babies are born these women appear less sensitive, responsive and more likely to show negative affectivity during interactions (Wellisch and Steinberg, 1980; Bauman and Dougherty, 1983; Fitzgerald et al., 1990; Burns et al., 1991, 1997; Rodning et al., 1991; Kelleher et al., 1994; Mayes et al., 1995; Chaffin et al., 1996; Ball et al., 1997; Swanson et al., 2000; Pajulo et al., 2001; Ukeje et al., 2001; Fraser et al., 2010; Salo et al., 2010; Eiden et al., 2011). Moreover, they appear less guiding and structuring (Blackwell et al., 1999; Pajulo et al., 2001; Salo et al., 2010), highly intrusive and intensely hostile (Rodning et al., 1991; Swanson et al., 2000; Johnson et al., 2002; Salo et al., 2009, 2010), showing, thus, difficulties in the main domains of adult emotional availability (Flykt et al., 2012). On the “younger” side, children of addicted mothers often show higher levels of irritability and difficulties in arousal regulation, which might compromise responsiveness to and involvement of the adult (Jeremy and Hans, 1985; Jacobson et al., 1996; Karmel et al., 1996; Mayes et al., 1996).

Considering “addicted parenting” from a transactional perspective (Sameroff and Fiese, 2000) it is possible to hypothesize that the child's difficulties might be exacerbated by a reduced capacity of the mother to function adequately as an interactive partner and as an external emotion regulator (Fogel, 1993; Lester and Tronick, 1994). This would lead to a subtle alteration of emotional processes and of the organization of mother-child interactions (Tronick et al., 2005). On the other hand, problems in emotion regulation might in turn interfere with the adult's ability to provide adequate caretaking.

Finally, a specific issue concerns the role of representational aspects on adult behaviors; it could be argued, in fact, that dysfunctional parenting practices might constitute a re-enactment of past life experiences. This hypothesis seems supported by the fact that these women often report histories of infantile trauma and abuse and frequently show high percentages of insecure or unresolved attachment representations (Simonelli and Vizziello, 2002; Caspers et al., 2006; Bakermans-Kranenburg and van Ijzendoorn, 2009; Stocco et al., 2012; Cassibba et al., 2013).

### Aim and hypothesis

The aim of this study was to monitor longitudinally parental abilities in four addicted women who presented four distinct patterns of adult attachment representations. More specifically, we aimed to investigate whether the development of dyadic patterns of interaction with their children varied on the basis of maternal attachment representations. The longitudinal perspective was included in order to focus more specifically on the processes underlining parental functioning and the organization of adult-child interactions. According to extant literature we hypothesized that:

- secure attachment representations in the mother would be associated with higher maternal sensitivity while insecure and unresolved attachment representations would be associated with lower maternal sensitivity.
- secure attachment representations in the mother would lead to better and more stable emotional exchanges, while mothers with insecure or unresolved attachment statuses would experience more difficulties and more changes during interactions with their children.

Moreover, we investigated whether there were specific indexes able to differentiate patterns of parenting between the two insecure mothers and the unresolved mother.

## Materials and methods

### Participants^1^

The study examined longitudinally four addicted women (*M* age = 19.75 years, *SD* = 5.12) and their children (two girls two boys) aged from 9 to 14 months (*M* = 11.25 months, *SD* = 2.22) at the beginning of the research. The subjects where extracted from a larger group of women following a rehabilitative program in a venetian Therapeutic Community (TC)^2^ specifically according to their attachment status and to the age of their children. All the subjects presented double diagnosis with borderline personality disorder.

Regarding substance abuse history, all mothers during the 12 months period before entrance in community showed a pathological pattern of abuse or use of substances which lead to a significant impairment or distress: for all of them, the main substance of abuse was heroin (4/4), followed by cocaine (4/4), cannabis (4/4), chemical drugs (3/4), methadone (3/4), hallucinogens (3/4), and medicines (1/4). The beginning of the rehabilitative program was partly chosen freely (2/4) partly consequence of a Juvenile Court decree (2/4). In all the cases the reason for entrance in TC was drug-addiction^3^. The mean age for the onset of the dependence was 13.75 years (*SD* = 0.479), whereas the intoxication period lasted on average 8.75 years (*SD* = 1.50). The onset of substance use was due partly to the escape of personal or familiar problems (3/4) and partly to the identification with the partner or the group of pairs (3/4). When the study took place all the women were subject to substitutive opioid treatment. Regarding their past life experiences only one subject reported familiarity for drug-abuse disorder. At admission only one woman still attended school, while the others had previously interrupted studies due to low socioeconomic status (1/4) or to substance abuse (2/4). At entrance all the mothers where unemployed. Some of them (2/4) experienced important losses or traumatic experiences (2/4) concerning physical, sexual or psychological maltreatment. Two of them were engaged in prostitution acts. With respect to pregnancy and motherhood, only one subject declared to have wished pregnancy. Two of them reported a continuative use of drugs during pregnancy. Regarding newborn's medical status at delivery, mean values were 38.75 weeks (*SD* = 1.258) for gestational age, 3.05 kilograms (*SD* = 0.385) for weight, 34 cm (*SD* = 1.155) for cranial circumference and 50.63 cm for length. Apgar scores at 1′ and 5′ ranged all between 9 and 10. Only one child showed signs of Neonatal Abstinence Syndrome (NAS) at delivery. As far as it concerns maternal medical history, three mothers presented Hepatitis C virus at admission.

### Procedure and instruments

#### Maternal attachment representations

Maternal attachment representations were assessed at entrance in TC with the Adult Attachment Interview (AAI—George et al., 1985). This semistructured interview aims to elicit information concerning an individual's current representation of his/her childhood experiences with the attachment figures. The interview consists of questions through which the participant is asked to recall and to reflect upon memories related to his/her attachment experiences with his/her caregivers during childhood. The AAI coding system allows to classify the individual into one of four attachment categories concerning adult attachment status: secure/autonomous (F), Dismissing (Ds), Entangled-Preoccupied (E), Unresolved with respect to a Loss and/or a Trauma (U). Individuals are classified as autonomous or secure (F) when they show coherence during the narration of their past attachment experiences, whether they were supportive or not. The dismissing (Ds) category is attributed when past experiences are described as too positive when compared to the actual content of narration. Dismissing individuals often complain that they are not able to recall past attachment-related memories. Individuals are classified as preoccupied (E) at the AAI when they show confused, angry or passive preoccupation with respect to their attachment figures. The excessive preoccupation given to their past memories may lead to the consequence that, when attending the interview, these subjects lose the focus from the context of discourse. Both dismissing and preoccupied subjects are considered insecure with respect to adult attachment representations. Moreover, individuals can be classified as unresolved/disorganized (U) regarding potentially traumatic experiences that concern loss or abuse. Indexes of non-resolved trauma are reflected through the momentary loss of the ability to monitor reasoning or the discourse. The interviews of the subjects had been previously independently rated by two raters who were unfamiliar with the sample and who had no access to demographic and psychiatric information. Both raters had been trained in conducting the coding and had substantial experience with the instrument. Inter-rater agreement was found to be excellent, with kappa ranging between 0.87 and 0.92.

#### Quality of mother-child interactions

Each 3 months during the stay in TC the dyads where observed and videotaped during 20-min free play sessions. The dyads of the study were observed for a total of seven periods (i.e., a longitudinal frame of observation that covers 21 months of stay in TC). Mother-child interactions where coded using the fourth version of the Emotional Availability Scales (EAS—Biringen, 2008). The construct refers to the ability of emotional sharing by taking part and contributing to a healthy and mutually fulfilling relationship (Biringen and Easterbrooks, 2012). It is composed of six scales/dimensions, four for the adult (sensitivity, structuring, non-intrusiveness, non-hostiliy) and two aimed at evaluating child behaviors (responsiveness, involvement of the adult). Each scale is composed of seven subscales.

*Adult sensitivity* refers to quality of adult affects, clarity of perceptions and appropriate responsiveness, awareness of timing, flexibility, variety and creativity during play, acceptance of the child, amount of interactions and adequate resolution of conflicts.
*Adult structuring* concerns the use of proactive guidance, the success of attempts, the amount of guidance, the ability to set limits and to remain firm in the face of pressure, the use of both verbal and non-verbal suggestions and the ability to assume an adult role rather than a peer one.
*Adult non-intrusiveness* refers to the ability to follow the child's lead, to the use of non-interruptive ports of entry into interaction, to the modest use of commands and directives, to the appropriateness of teaching and adult talking, to the absence of interferences and of child's signals that indicate that the adult is perceived as intrusive.
*Adult non-hostility* refers to the lack of negativity in face or voice and to the lack of ridiculing or other disrespectful behaviors toward the child. A non-hostile adult does not threat to separate, is not frightening, maintains cool during challenging situations and does not use threats of hostile play themes during interactions.
*Child responsiveness* takes into account quality of child's affects and organization of behaviors, the ability and the willingness to respond to the adult's bids without anxiety or role reversal. It also considers positive physical positioning, concentration on task and the presence of avoidance or of over responsiveness and role reversal.
*Child involvement of the adult* concerns the use of simple and elaborative initiative to involve the adult, the affective use of the adult (rather than instrumental), the lack of negative/over involving behaviors and the use of verbal and non-verbal channels.

Each EA dimension is given a global score on a 7 point scale, where higher ratings stand for more optimal features. Values between 5 and 7 are representative of an emotionally available dyad and considered index of a healthy relationship. Scores around 4 indicate complicated emotional availability, that is behaviors that are appropriate in some ways but that are not fully healthy (for example a mother apparently sensitive or inconsistent in structuring). Scores around 3 indicate less optimal aspects (for example a caregiver who is somewhat insensitive, intrusive or slightly overtly hostile) while the range between 1 and 2 concerns more problematic behaviors (i.e., a mother extremely insensitive, withdrawn or aggressive, extreme role reversal or the presence of disorganization in child's affects and behaviors)^4^. To get a more detailed profile of the interaction, the observer can also attribute a score to the seven subscales of each dimension. The first two subscales get scores from 1 to 7, while the other five a score between 1 and 3. According to the EA coding system (Biringen, 2008), scores are considered adequate when they are above the mid-point of the scale, inconsistent when they coincide with it, and inadequate when they are below it. The interactions were rated by two coders trained on the EAS system who were blind with respect to the attachment status of the participants.

## Results

### Adult attachment representations and patterns of mother-child interactions

As previously said, the mothers were extracted from a larger group in accordance with their adult attachment status, in order to compare the four main AAI categories with respect to the evolution in time of mother child-interactions.

Table 1 shows average scores and standard deviations for each dyad on the 6 EA dimensions. With respect to adult scales, the autonomous (F) and the dismissing (Ds) mother are the subjects that presented better parenting qualities, reporting average scores above the mid-point on each EA adult dimension. The preoccupied (E) and the unresolved (U) mother instead seemed to experience more difficulties during mother-child interactions; they appeared less sensitive and less able to structure and to guide emotional exchanges. Moreover, the unresolved (U) mother resulted more intrusive and less able to regulate tensions and negative emotions, reporting scores below 4 also on the scales of non-intrusiveness and non-hostility. Considering the child variables, again children of the secure (F) and of the dismissing (Ds) mother appeared to enjoy more positively interactions with their caregivers, showing scores above average both on child responsiveness and on child involvement of the adult. On the other side, children of the preoccupied (E) and of the unresolved (U) mother, seemed to experience more difficulties. While the latter one seemed to oscillate around average, reporting scores around 4, the first one exhibited more difficulties, resulting more avoiding and less engaged in emotional exchanges with his mother.

**Table 1:** **Average scores and standard deviations of mother-child interactions assessed with the Emotional Availability Scales (EAS)**.

**EA dimension**		**Secure (F) *M* (*SD*)**	**Dismissing (Ds) *M (SD)***	**Preoccupied (E) *M (SD)***	**Unresolved (U) *M (SD)***
Sensitivity	Global score	6.36 (0.476)	4.29 (1.075)	3.71 (0.393)^**^	3.929 (0.189)^**^
	Affects	6.57 (0.535)	4.14 (1.029)	3.64 (0.378)^**^	4.00 (0.000)^*^
	Perception e Responses	6.43 (0.535)	4.50 (1.658)	3.79 (0.699)^**^	3.64 (0.556)^**^
	Timing	2.86 (0.378)	2.36 (0.441)	1.86 (0.244)^**^	1.93 (0.283)^**^
	Flexibility	2.79 (0.393)	2.36 (0.441)	1.86 (0.244)^**^	2.07 (0.450)
	Acceptance	3.00 (0.000)	2.14 (0.408)	2.57 (0.627)	1.57 (0.463)^**^
	Amount of interaction	3.00 (0.000)	2.50 (0.408)	2.36 (0.627)	2.71 (0.488)
	Conflicts	2.71 (0.488)	2.86 (0.378)	2.57 (0.787)	2.29 (0.488)
Structuring	Global score	5.86 (0.748)	4.71 (1.577)	3.71 (0.488)^**^	3.86 (0.378)^**^
	Guidance	6.21 (0.698)	4.50 (1.555)	3.79 (0.393)^**^	3.93 (0.476)^**^
	Success	6.14 (1.069)	5.07 (1.835)	3.57 (0.976)^**^	3.86 (0.476)^**^
	Amount of structure	2.86 (0.378)	2.29 (0.393)	2.14 (0.378)	2.86 (0.378)
	Limit setting	2.57 (0.535)	2.57 (0.535)	2.14 (0.378)	2.00 (0.000)^*^
	Remaining firm	2.86 (0.378)	3.00 (0.000)	2.29 (0.756)	2.29 (0.488)
	Verbal vs. non-verbal	2.86 (0.378)	2.43 (0.535)	2.00 (0.577)^*^	2.50 (0.627)
	Peer vs. adult role	2.64 (0.476)	2.29 (0.393)	1.93 (0.189)^**^	1.79 (0.636)^**^
Non-intrusiveness	Global score	5.07 (0.838)	5.71 (1.150)	4.00 (0.957)^*^	2.86 (0.852)^**^
	Following child's lead	5.00 (0.816)	5.71 (0.951)	4.07 (0.951)	3.36 (0.951)^**^
	Ports of entry	5.43 (0.535)	6.00 (1.414)	3.86 (1.069)	2.57 (0.787)^**^
	Commands	2.64 (0.476)	2.43 (0.535)	2.00 (0.289)^*^	1.79 (0.393)^**^
	Talking	2.71 (0.488)	2.86 (0.378)	2.29 (0.488)	2.43 (0.535)
	Teaching	2.00 (0.000)^*^	2.71 (0.488)	2.07 (0.450)	2.00 (0.577)^*^
	Interferences	2.57 (0.535)	2.64 (0.748)	2.14 (0.900)	1.14 (0.378)^**^
	Child's feedback	2.86 (0.378)	2.86 (0.378)	2.29 (0.756)	1.93 (0.607)^**^
Non-hostility	Global score	6.43 (0.732)	5.36 (1.029)	5.29 (1.075)	3.71 (0.951)^**^
	Negativity	6.29 (0.756)	5.14 (1.345)	5.29 (1.113)	3.50 (1.000)^**^
	Ridiculing	6.86 (0.378)	6.00 (1.155)	5.57 (1.272)	3.71 (0.951)^**^
	Threats of separation	3.00 (0.000)	3.00 (0.000)	3.00 (0.000)	2.86 (0.378)
	Lose cool	2.71 (0.488)	2.71 (0.488)	2.29 (0.488)	1.79 (0.267)^**^
	Frightening	3.00 (0.000)	2.50 (0.500)	3.00 (0.000)	2.71 (0.488)
	Silence	3.00 (0.000)	2.50 (0.500)	3.00 (0.000)	2.86 (0.378)
	Hostile play themes	3.00 (0.000)	2.93 (0.189)	3.00 (0.000)	3.00 (0.000)
Child Responsiveness	Global score	6.07 (1.170)	5.00 (1.225)	3.29 (0.567)^**^	4.00 (0.500)^*^
	Affects	6.14 (1.215)	5.14 (1.376)	3.00 (0.707)^**^	4.00 (0.500)^*^
	Responsiveness	5.86 (1.676)	5.29 (1.254)	3.50 (0.408)^**^	3.93 (0.607)^**^
	Autonomy	2.86 (0.378)	2.43 (0.535)	1.79 (0.636)^**^	2.29 (0.488)
	Physical positioning	2.57 (0.787)	2.43 (0.607)	1.64 (0.476)^**^	2.29 (0.488)
	Role reversal	3.00 (0.000)	2.71 (0.488)	3.00 (0.000)	3.00 (0.000)
	Avoidance	2.86 (0.378)	2.79 (0.576)	1.79 (0.393)^**^	2.43 (0.535)
	Task orientation	2.93 (0.189)	2.57 (0.607)	1.71 (0.393)^**^	2.00 (0.000)^*^
Child Involvement	Global score	5.86 (1.180)	5.14 (1.215)	3.29 (0.809)^**^	3.86 (0.476)^**^
	Simple initiative	6.00 (1.414)	5.50 (1.190)	3.36 (0.748)^**^	4.00 (0.577)^*^
	Elaborative initiative	5.86 (1.215)	4.71 (1.254)	3.14 (0.900)^**^	3.57 (0.787)^**^
	Use of adult	3.00 (0.000)	2.86 (0.378)	1.71 (0.488)^**^	2.29 (0.488)
	Over-involvement	2.43 (0.535)	2.71 (0.488)	2.21 (0.994)	2.07 (0.732)
	Eye contact	2.86 (0.378)	2.57 (0.535)	1.93 (0.189)^**^	2.43 (0.535)
	Body positioning	2.86 (0.378)	2.71 (0.488)	2.14 (0.627)	2.57 (0.535)
	Verbal involvement	3.00 (0.000)	2.86 (0.378)	2.07 (0.732)	2.71 (0.488)

* *Scores on the mid-point of the scale (i.e., inconsistent)*.

** *Scores below the mid-point of the scale (i.e., at risk)*.

**Figure 1:**
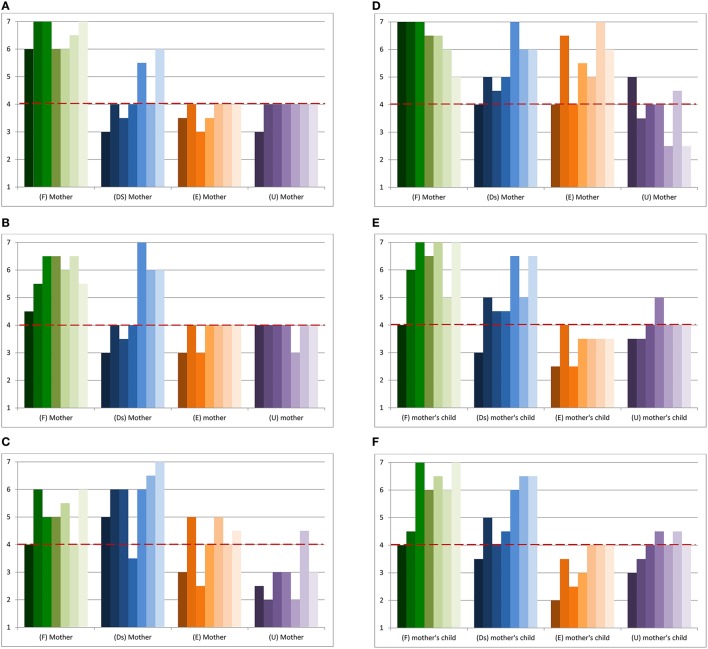
**(A)** Longitudinal ongoing of maternal sensitivity in the four dyads. **(B)** Longitudinal ongoing of maternal structuring in the four dyads. **(C)** Longitudinal ongoing of maternal non-intrusiveness. **(D)** Longitudinal ongoing of maternal non-hostility. **(E)** Longitudinal ongoing of child responsiveness. **(F)** Longitudinal ongoing of child involvement of the adult. The dotted red lines on the mid-point of the scale discriminate between the optimal and the non-optimal zone in the EA dimension considered.

Figure 1A shows the longitudinal ongoing of maternal sensitivity in the four dyads considered. As it is possible to see, during all the episodes taken into account the secure (F) mother proved to be adequately sensitive and responsive toward her child's cues, showing a good and spontaneous quality of affects. The dismissing (Ds) mother, instead, seemed initially to get scores on the mid-point (T2, T4) or below it (T1, T3) while showing later an increase in the scores (T5, T7), alternated with a periodical way back on the mid-point (T6). As far as it concerns the preoccupied (E) mother, the woman oscillated from being inconsistently sensitive (T2, T5, T6, T7) to being moderately insensitive toward her child's cues (T1, T3, T4). Finally, with the exception of the first episode (T1), the unresolved (U) mother appeared inconsistently sensitive, getting always scores around four on this dimension.

Figure 1B represents the longitudinal ongoing of maternal structuring. As shown by the graphs, the secure mother (F) proved to be successful in guiding interactions during all the periods considered, whereas the preoccupied (E) and the unresolved (U) mother never got scores above average; in other words, these women seemed to make a lot of attempts in trying to guide that however were not successful, maybe because they were not tuned on the children's interests or maybe because the attempts were too much and somehow they “lost” their children, speaking in terms of affectively and attentively sharing. Regarding the dismissing (Ds) dyad, initially the mother tended to get scores on the mid-point or below, while from T4 this ability seemed to grow.

Figure 1C reports the longitudinal ongoing of maternal non-intrusiveness during the periods considered. As it is possible to notice from the graphs, with the exception of some cases where she appeared benignly intrusive (T4 and T6), the secure (F) mother resulted able to leave enough space to the child without interfering with her activities during mother-child interactions. The same considerations can be made regarding the dismissing (Ds) mother who, globally, did not appear intrusive during the sessions considered, with the exception of one episode (T4). As happened for structuring, the preoccupied (E) mother seemed to oscillate in non-intrusiveness too; in this case she alternated moments where she appeared non-intrusive, i.e., able to leave enough space to the child (T2, T5), and moments where she resulted moderately intrusive (T4, T6) or definitely intrusive (T1, T3) toward the child's activities. Finally, with the exception of an episode (T6), the unresolved (U) mother appeared constantly over-suggestive and interfering during free play interactions with her child.

Figure 1D reports the longitudinal ongoing of maternal non-hostility. As shown by the graphs the mothers with secure (F), dismissing (Ds) and preoccupied (E) attachment statuses appeared globally non-hostile; with the exclusion of few episodes where signs of covert hostility were noticed, these women appeared consistently able to downregulate their negative affectivity (i.e., the scores on this scale never got below the mid-point). In the unresolved (U) dyad, instead, it is possible to notice different times where the adult did not show optimal modulation of negative affects (T2, T5, T7), expressing overt hostility during play with her child.

Figure 1E shows longitudinally patterns of child responsiveness in the four dyads. A first consideration can be made on the child of the secure (F) mother; as reported by the figure, except for an initial moment (T1) where she seemed to show some slight difficulties in responding to her mother's bids, from the second period on the scores in this scale increased, indicating thus the reach of an adequate organization of affects and responding behaviors. Concerning the dismissing (Ds) dyad, at the beginning of the program the child showed non-optimal responsiveness, whereas this feature increased later in time. As it is observable, the child of the preoccupied (E) mother did not show optimal responsiveness during interactions, appearing rather avoiding and getting, with few exceptions, scores below the mid-point of the scale. Finally, the child of the unresolved (U) mother seemed to move from non-optimal (T1, T2) to complicated responsiveness (T5, T6, T7) with a slight increase in the scores during T4.

Finally, Figure 1F represents the longitudinal ongoing of child involvement of the adult. As reported from the graphs, after some initial difficulties, the child of the secure (F) and of the dismissing (Ds) mother seemed able to and successful in involving their parents during free play interactions, receiving almost every time scores above the mid-point. The other two dyads showed more difficulties on this EA dimension, especially the child of the preoccupied (E) mother, who initially appeared consistently avoidant during videotaped sessions. The child of the unresolved (U) mother, instead, showed more frequently scores around, or slightly above, the mid-point of the scale.

## Discussion

This study aimed to assess parenting of drug addicted mothers longitudinally, from different perspectives. Two of the main components of parental functioning were considered: maternal representations with respect to early attachment experiences and actual parenting behaviors enacted during adult-child interactions. To assess adult attachment representations we adopted the AAI (George et al., 1985) while mother-child interactions were evaluated through the EAS (Biringen, 2008), a tool that originated in part from attachment theory but that also tried to enlarge the focus of investigation integrating contributions from emotion, systemic and transactional theories. In this sense we tried to enrich the attachment perspective with an interactive and relational one, in order to understand more deeply how parenting and adult-child relationships might influence each other. Moreover, adult-child interactions were assessed longitudinally, during the first 21 month of stay in the facility; in this sense, the inclusion of a longitudinal perspective allowed to expand knowledge on the dynamic processes underlying parental functioning and the organization of adult-child interactions, rather than to rely on a single and more static evaluation of them. The four mothers were chosen according to their adult attachment status assessed at admission in TC, in order to collect one subject for each of the four AAI main categories, that is secure/autonomous (F), dismissing (Ds), entangled-preoccupied (E) and unresolved with respect to trauma and/or losses (U). Quality of mother-child interactions, instead, was considered longitudinally and assessed each 3 months during the first 21 months of stay in the facility.

We hypothesized secure attachment representations in the mother to be associated (a) with higher maternal sensitivity and (b) with more stable emotional exchanges if compared to the insecure or the unresolved ones. As expected, the results highlighted marked differences in interactive competencies in the secure vs. insecure/unresolved mothers. More specifically the dyad with the secure (F) mother presented interactions of better quality, with the mother being more sensitive and responsive toward her child's cues and the child showing more optimal and positive responsiveness and involvement of the adult; moreover, these results appeared predictable over time (i.e., the scores always remained in the optimal range or above the mid-point of the scales). On the other side, the insecure (Ds) (E) dyads and the one with the unresolved (U) mother presented more changeable patterns of interactions and more difficulties during the periods observed, with the mothers resulting inconsistent in the ability to adequately perceive and respond to their infants' signals.

In this sense, with respect to our initial hypothesis we can conclude that:

- secure attachment representations in the mother resulted associated with higher maternal sensitivity during mother-child interactions while mothers with insecure and unresolved attachment representations presented lower degrees of maternal sensitivity.
- the dyad with the secure mother experienced better emotional exchanges, that appeared more stable in time (i.e., always above the mid-point of the scale) while the dyads with the insecure mothers or with the unresolved mother showed more difficulties and more “jagged” patterns of mother-child interactions.

A first consideration about our study concerns the particular group of subjects taken into account, that is drug addicted women following a therapeutic rehabilitation program in a residential community. On one side, in fact, these women presented the typical risk factors often associated with drug dependence (i.e., past experiences of traumas and losses, low socioeconomic status and low levels of education). Moreover, all the subjects presented double diagnosis with borderline personality disorder, a condition often associated with particularly detrimental interactive features on the maternal side, such as rapid changes from sensitive to punishing responses (the so-called “*oscillations in parenting*”), emotion dysregulation and general distress (Fruzzetti, 2012; Stepp et al., 2012). On the other hand these mothers also benefited, at least in part, from the therapeutic and the educational interventions offered by the community staff, which could have worked as protective factors for parenting and interactions instead. In this sense, when discussing the results of our study it is important to keep in mind both the risk factors associated with the condition of drug addiction and the moderating role played by TC as a “buffer” on the quality of caregiving.

Another consideration should be addressed specifically to the need of assuming a multi-level perspective when assessing parenting (De Palo et al., 2014). One of the major aspects emphasized by attachment theorists, in fact, concerns maternal sensitivity and responsivity, the two main ingredients considered as necessary to fulfill a secure attachment relationship (Ainsworth, 1969; Bowlby, 1984). It has been hypothesized that maternal sensitivity could work as a mediating factor between parental attachment representations and internal working models of their children (van Ijzendoorn and Bakermans-Kranenburg, 1999). It has been suggested that parents secure with respect to past attachment experiences would be more likely to enhance secure relationships with their children, thanks to their ability to correctly read and interpret infant signaling (Main, 1990). On the other hand, past experiences of insecure parents might compromise somehow their opennes to infant bids, interfering with the quality of emotional interactions (van Ijzendoorn and Bakermans-Kranenburg, 1999). These hypothesis have been in part confirmed by empirical research (Aviezer et al., 1999, 2003; Ziv et al., 2000; Sagi et al., 2002; Biringen et al., 2005; Aviezer, 2008; Easterbrooks et al., 2012), although sometimes the results were less clear and linear (Aviezer et al., 1999, 2003; Biringen et al., 2005, 2014). The modest effect size found for the link between adult attachment status determined by the AAI and sensitivity, and for the link between the latter and the quality of child attachment, suggested a “transmission gap,” where other variables could intervene in determining the intergenerational transmission of attachment (Sagi et al., 1994; Van Ijzendoorn, 1995; van Ijzendoorn and Bakermans-Kranenburg, 1999; Martins et al., 2012; Van den Dries et al., 2012; Biringen et al., 2014). Multidimensional observational and assessment tools appear thus particularly useful, since they combine the assessment of sensitivity with the focus on other aspects of adult behavior, allowing to reach a more comprehensive vision of parental functioning. Effectively, considering our results (but also keeping in mind that our work concerned a “special” population), we may say that sensitivity seemed only sufficient to differentiate parenting of the secure mother from the parenting of the insecure and the unresolved mothers considered as a group (i.e., the insecure and the unresolved mothers did not show marked differences from each other in this EA dimension). As a consequence we did not find a clear pattern of adult sensitivity specifically related to each adult attachment status and able to discriminate between non-secure AAI categories.

The same considerations could be made on adult structuring, given that both the insecure mothers and the unresolved one showed general difficulties in adequately guiding and scaffolding their children during mother-child interactions without presenting a specific and predictable pattern of inconsistency.

Differently from the secure and the dismissing mother, the preoccupied and the unresolved mother appeared rather intrusive during the periods considered. Women with this AAI status have been previously described as over-involved, over-stimulating, unpredictable and affectively deregulated toward their infants (De Palo et al., 2014). Moreover, intrusive behaviors have been often reported as a specific feature that characterizes parenting of addicted women (Rodning et al., 1991; Johnson et al., 2002; Salo et al., 2009, 2010). In other studies maternal intrusiveness showed higher associations with avoidant and disorganized patterns of attachment (Swanson et al., 2000). Maternal intrusiveness seemed thus to constitute here a better discriminating factor, since it appeared specific of only two AAI non-secure categories.

Finally, maternal non-hostility appeared to be the only dimension of our study able to differentiate between the interactive pattern of the unresolved mother and the others (the secure and the insecure ones). This woman, in fact, showed persistent difficulties in modulating negative affectivity during the interactions with her child. Negative affectivity and hostility have been pointed out in the literature as particularly adverse factors with respect to child development and mother-child interactions. Some authors (Main and Hesse, 1990) suggested frightening maternal behaviors to be more likely to lead to a disorganization of the attachment strategies in the child, due to the paradox of searching for protection from fear into a fearful adult. Other authors (Beebe and Lachmann, 2002), instead, focused more specifically on the detrimental effects of continuous interactive-ruptures not followed by repair, that in our case might be determined by the continuous expressions of negative affect from the mother.

Resuming our results, we can conclude that, in part, in our study we have noticed some of the difficulties that the literature has often associated to parenting of addicted mothers. On the other hand we have also observed, as already reported in different studies, that, despite the condition of drug addiction, having secure attachment representations is more likely to be associated with more positive adult-child emotional exchanges. Globally, these results seem to direct us toward the road of complexity when assessing parental functioning, underlining the need to consider different and less “traditional” aspects of the caregiver besides the more studied ones, and to integrate them in a more comprehensive vision of the individual considered as a caregiver (Espinet et al., 2013). Furthermore, this study can be considered part of a more general effort made to enhance the integration of research and clinical thinking, in order to create relational profiles specific for each dyad, considering both individual and dyadic functioning, with the purpose to establish personalized and targeted interventions. In line with this, recently some authors highlighted the importance of integrating an attachment-relational perspective with a multi-level assessment of individual functioning when planning interventions for parents in clinical or at-risk populations, in order to reduce the risks of failure (De Palo et al., 2014).

Of course the data presented in this paper cannot be generalized to the wider population, especially given the case study nature of our work. First of all this is due to the particular group of subjects considered; the fact that the TC might intervene as a buffering factor could exert a confounding effect, preventing us from asserting with certainty that the difficulties found in our dyads are the same that other dyads with addicted parents non-under treatment might experience (the latter in fact might be more severe). The same could be said concerning the change in time of emotional availability (for example, it is not said that any other individual with dismissing adult attachment representations would undergo the same improvements that the (Ds) mother of our study experienced). Secondly, a generalization of these results is not possible given the small amount of subjects considered and the absence of a control group; on one hand, in fact, the absence of a comparison croup does not allow to control the eventuality that the attachment style and the condition of drug addiction interacted together creating a unique style of mother-infant interaction; moreover, the presence of gender differences in each dyad might have somehow influenced the relational style, so that absolute conclusions cannot be drawn from the comparison of the four attachment styles. Anyway, despite these limits, these data seem to be in line with a large amount of theoretical and empirical literature on attachment, parenting, and early mother-child interactions, confirming in part the differences expected between dyads with secure mothers and dyads with insecure or unresolved mothers (van Ijzendoorn and Bakermans-Kranenburg, 1999; Ziv et al., 2000; Sagi et al., 2002; Aviezer, 2008; Easterbrooks et al., 2012). From this point of view they appear a promising starting point for future research; in fact, more standardized research designs applied to larger samples, and with comparisons between clinical and non-clinical-subjects, would allow a better comprehension of common or specific/individual trajectories regarding both parenting and child development in risk- and non-risk groups. Moreover, the comparison between dyads in treatment and without treatment would lead to a deeper understanding of the intervening mechanisms with respect to efficacy of therapeutic programs; this in fact is an aspect that our study did not take enough in consideration. Finally, an objective for future research should be a greater focus on the contribution of children and of their intrinsic features. It appears in fact always more clear how the child could intervene as an active partner in shaping the quality of the relationship and the development of parental functioning, especially in the presence of peculiar features that are present since from delivery.

## Author contributions

AS prepared the study design, supervised the research team, and wrote the discussions section of the manuscript; NC organized the recruitment of the sample and supervised data collection; AP and FD wrote the introduction section and the references, prepared data set, performed statistical analyses and prepared tables and figures; All authors reviewed the manuscript.

### Conflict of interest statement

The authors declare that the research was conducted in the absence of any commercial or financial relationships that could be construed as a potential conflict of interest.

## Footnotes

^1^This study was carried out in accordance with the recommendations of the Code of Ethics approved by the General Assembly of the Italian Association of Psychology held on March 2015 the 27th. The data presented in this paper constitute part of a larger research project approved in July 2014 by the Ethical Committee of the University of Padua (code. 1444).

^2^Therapeutic Community for drug-addicted women and their children offers residential care to the mother-child dyad and provide a comprehensive rehabilitation program, which takes place during a 2-year stay. The access in the community for addicted mothers and their children is applied by a decree of the Juvenile Court and implies a coercive intervention for the mother; the risk is to be immediately separated from her child (Stevens et al., 1989; National Institute on Drug Abuse). As for the intervention methods, a combined treatment (i.e., for both parent and child together) is carried out on an intensive basis (the dyads are in residential care): TCs typically offer a therapeutic program, which is centered on the individual-parent-child system taken as a whole (Meisels et al., 1993).

^3^Sometimes the rehabilitative program might be an alternative from jail or due to psychiatric status.

^4^When referring to the global scores, the term “around” is used to indicate the different clinical shades that a certain score might assume. The scoring system, in fact, offers specific anchor points (i.e., 3; 4; 5; and so on) with prototypical descriptions, but allows at the same time to use half points to emphasize specific features of the observed interaction (for example, a score of 3, 5 could be used to indicate an interaction lacking of emotional warmth and mother who is mechanical and somewhat insensitive but that, at the same time, shows a lot of efforts to succeed with the child; in this sense, the mother would be equally considered as somewhat insensitive, getting indeed a score “around 3,” but the half point would allow to keep in mind that the quantity of efforts done, although not enough to increase the score to a full 4, are higher than a typical 3.
